# In-home dementia caregiving is associated with greater psychological burden and poorer mental health than out-of-home caregiving: a cross-sectional study

**DOI:** 10.1080/13607863.2021.1881758

**Published:** 2021-02-08

**Authors:** S. Brini, A. Hodkinson, A. Davies, S. Hirani, R. Gathercole, R. Howard, S. P. Newman

**Affiliations:** a School of Health Sciences, City, University of London, London, UK; b National Institute for Health Research (NIHR) School for Primary Care Research, Manchester Academic Health Science Centre, University of Manchester, Manchester, UK;; c Centre for Academic Child Health, University of Bristol, Bristol, UK; d Division of Psychiatry, University College London, London, UK

**Keywords:** Caregiver burden, mental health, dementia, informal caregiver, psychological strain

## Abstract

**Introduction:**

Caregivers who live with a person with dementia who receives care, compared with those who live elsewhere, are often considered to experience greater levels of psychological and affective burden. The evidence for this is, however, only limited to studies employing small sample sizes and that failed to examine caregivers’ psychological wellbeing. We address these issues in a large cohort of dementia caregivers.

**Methods:**

We conducted a cross-sectional study comparing caregivers living with a dementia care recipient (*n* = 240) to caregivers living elsewhere (*n* = 255) on caregivers’ burden, anxiety, and depression.

**Results:**

We found that caregivers living with the care recipient relative to those living elsewhere showed significantly greater burden and depression, but we found no group difference in anxiety.

**Conclusions:**

Our study adds to the evidence by showing that cohabiting with a care recipient with dementia is associated with greater burden and poorer psychological wellbeing. Strategies aiming to improve caregivers’ burden and psychological wellbeing should take account of caregivers’ living arrangements.

## Introduction

### Background

There were approximately 43.8 million people living with dementia in 2016 worldwide (Nichols et al., 2019) with 115.5 million people expected to develop dementia by 2050 (Prince et al., 2013). The total societal worldwide costs of dementia per year reached US$1 trillion in 2018 with informal caregiving accounting for US$1057 billion of that total cost (Wimo et al., 2017). Informal caregivers of people living with dementia are often family members, without whom the care recipient would likely experience poorer health outcomes (Brodaty & Donkin, 2009). Providing informal care by helping with basic and instrumental activities of daily living and time spent in supervision to individuals with dementia (Wimo et al., 2017) places considerable psychological strain on the caregiver (Cooper, Balamurali, & Livingston, 2007; van der Lee, Bakker, Duivenvoorden, & Dröes, 2014). Caregiver burden, in turn, can negatively influence health outcomes in the care recipient with dementia (Stall et al., 2019). The World Health Organization has described informal caregiving as a key issue in dementia care (WHO, 2012).

There is currently no official definition of caregiver burden (Adelman, Tmanova, Delgado, Dion, & Lachs, 2014), although it typically refers to the caregiver perceiving his or her caregiving role as negatively impacting their own affective and psychological functioning (Zarit, Todd, & Zarit, 1986). Caring for someone with dementia places greater psychological burden on the caregiver (Sallim, Sayampanathan, Cuttilan, & Ho, 2015) than caring for individuals with other chronic conditions (Loh, Tan, Zhang, & Ho, 2017). The observed increase in caregiver burden can result in earlier institutionalization for care recipients with dementia (Balardy, Voisin, Cantet, & Vellas, 2005; Brodaty & Donkin, 2009), particularly when the caregiver feels ‘trapped’ in caregiving duties (Gaugler, Yu, Krichbaum, & Wyman, 2009). Caregivers for people with dementia also experience poor physical health (Gilhooly et al., 2016) and mental health (Cuijpers, 2005; Richardson, Lee, Berg-Weger, & Grossberg, 2013; Watson, Tatangelo, & McCabe, 2019). Psychosocial strategies are sometimes unsuccessful in improving caregivers’ health outcomes (Cooke, McNally, Mulligan, Harrison, & Newman, 2001; Vandepitte, Van den Noortgate, Putman, Verhaeghe, Faes, et al., 2016; Vandepitte, Van Den Noortgate, Putman, Verhaeghe, Verdonck, et al., 2016) because these strategies often discount contextual factors such as whether the caregiver lives with the care recipient or elsewhere (Vandepitte, Van Den Noortgate, Putman, Verhaeghe, Faes, et al., 2016; Vandepitte, Van Den Noortgate, Putman, Verhaeghe, Verdonck, et al., 2016).

While having a co-resident caring for someone with dementia may delay institutionalization (Banerjee et al., 2003), caregivers living with the care recipient experience greater psychological burden than caregivers living elsewhere (Conde-Sala, Garre-Olmo, Turró-Garriga, Vilalta-Franch, & López-Pousa, 2010; Kim, Chang, Rose, & Kim, 2012; Raccichini, Castellani, Civerchia, Fioravanti, & Scarpino, 2009; Reed et al., 2014). This may not always be the case for quality of life, however (Tay et al., 2016). The observed higher burden and poorer mental health among caregivers cohabiting with the care recipient with dementia increases over time (Viñas‐Diez et al., 2017). The limited number of studies exploring the link between cohabitation and caregivers’ burden have employed relatively small sample sizes (Conde-Sala et al., 2010; Raccichini et al., 2009; Tay et al., 2016) and did not specifically examine anxiety or depression as outcomes, both of which are highly prevalent among caregivers of people with dementia (Cuijpers, 2005; Richardson et al., 2013; Watson et al., 2019). These studies also did not explore whether different dimensions of the burden scale that was administered might be differentially affected by caregivers’ living arrangements (Conde-Sala et al., 2010; Raccichini et al., 2009; Reed et al., 2014; Tay et al., 2016).

### Objectives

In this study, we extend previous research (Conde-Sala et al., 2010; Raccichini et al., 2009; Reed et al., 2014) by using a larger sample of participants from the Assistive Technology and Telecare to maintain Independent Living At home for people with dementia (ATTILA) trial (Leroi et al., 2013). We investigated whether caregivers’ living status is related to burden, anxiety, and depression. The primary objective of the study was to examine whether caregivers living together with the care recipient with dementia experienced greater burden and poorer mental health than caregivers not living with the care recipient. We hypothesized that caregivers living with the care recipient would demonstrate greater burden than caregivers not living together with the care recipient. We also hypothesized that caregivers living with the care recipient would demonstrate greater depression and anxiety levels than caregivers not living with the care recipient

## Methods

This cross-sectional study was conducted in accordance with the guidelines for Strengthening the Reporting of Observational Studies in Epidemiology (STROBE) statement: guidelines for reporting observational studies (Von Elm et al., 2014).

### Study design

We conducted a between-group cross-sectional study to explore whether caregiver status (live-in *vs.* live-out) is associated with different levels of caregiver burden, anxiety, and depression. The predictor was caregivers’ living status with two levels: (1) whether the caregiver lived together with the care recipient with dementia or (2) lived elsewhere. The outcomes included caregiver burden, depression, and anxiety. We examined caregivers’ data from the ATTILA trial dataset, including caregivers’ cohabitation status, age, burden, anxiety, and depression as well as the care recipients’ degree of cognitive impairment. The description of the full ATTILA trial design is reported elsewhere and is a randomized controlled trial (Trial Protocol Reference ISRCTN86537017) to assess the impact of telecare technologies on the move to institutionalized care and caregiver outcomes (Leroi et al., 2013).

### Setting

Participants were recruited through the National Health Service and from local social services in the United Kingdom (Leroi et al., 2013) to participate in a pragmatic randomized-controlled trial over 104 weeks from January 2013 for 4 years.

### Participants and outcomes

The data analysed in this study include the secondary outcomes of the caregiver of each care recipient with dementia that was collected as part of the data collection process to address the primary aim of the ATTILA trial (Leroi et al., 2013). Therefore, participants were the informal (unpaid) caregivers of participants recruited to the main ATTILA randomized controlled trial (Leroi et al., 2013). Participants in the trial were: caregivers of individuals with dementia (young or later-onset) or those who presented evidence of cognitive difficulties deemed to be sufficient to indicate dementia with or without a formal diagnosis, both with and without capacity. The participants described here were their respective caregivers, aged 18 years or above, including spouses, partners, or children of the dementia care recipient. The caregivers remained part of the ATTILA trial or until their care recipient was institutionalized or died (Leroi et al., 2013).

### Variables

Demographic variables relating to the caregiver such as age, caregiver living status, caregiver relationship to the cared-for person (spouse/partner, child/child-in-law, other relative, and non-relative) and the Standardized Mini-Mental State Examination (SMMSE) to determine the degree of cognitive impairment in the caregiver were collected. To assess caregiver burden and psychological functioning, we examined participants on the 22-item Zarit Burden Inventory (ZBI), anxiety using the 6-item State-Trait Anxiety Inventory (STAI-6), and depression with the 10-item Centre for Epidemiological Studies Depression Scale (CES-D-10). In addition to the total score on the ZBI, we also generated three subscales using principal component analysis (PCA). These were negative appraisal caring (caregivers negatively assessing their role), adequacy (whether caregivers felt adequate in providing care), and burden strain (caregivers estimating the level of strain from providing care). Higher scores in each measure indicate greater burden and poorer mental health. Each measure demonstrates good psychometric properties (Bachner & O'Rourke, 2007).

### Data sources

While the ATTILA trial was longitudinal, for this study we analysed baseline caregivers’ data, collected at week 0, prior to implementing the intervention.

### Bias

Since this study is a secondary analysis of the caregivers of the primary participants in the ATTILA trial, control of potential biases was handled in consideration of the care recipients rather than their caregivers. Pre-planned analyses for bias in the ATTILA trial are described elsewhere (Leroi et al., 2013). However, we planned a sensitivity analysis of missing data after imputation.

### Study size

The sample size was based on the ATTILA trial’s primary outcome i.e. time to institutionalization (Leroi et al., 2013). Therefore, for this secondary analysis, the sample size was not informed by caregivers’ outcomes.

### Quantitative variables

Participants were grouped between those who had reported living together with the care recipients (live-in) and those who had reported not living with the care-recipient (live-elsewhere).

### Statistical methods

All analyses were conducted in R statistical software using the lme4 and lavaan packages. An alpha level of .05 was applied. For each demographic factor and outcome described under the *Variables* section, we presented means and standard deviations (SD) as well as 95% confidence intervals (CI). We also reported the median and interquartile range (IQR) for the sensitivity analysis. To explore the relationship between caregivers’ status (live-in *vs.* live-elsewhere), we conducted a series of Linear mixed-effects (LME) models on each outcome (burden, depression, and anxiety).

### Missing data

Multiple imputation was used to obtain a more complete data set and to better protect against bias due to missing data. In the unimputed dataset, the percentage of missing data was below 23% in any of the individual demographic variables or outcomes. Missing values for age, SMMSE, and all three outcome scores were imputed using the Multivariate Imputations *via* Chained Equations approach using the packages in R (https://cran.r-project.org/web/packages/mice/mice.pdf). This process produces several data sets, each of which is analysed separately using the prespecified model; the results are then combined while accounting for uncertainty in imputed values (Donders, Van Der Heijden, Stijnen, & Moons, 2006; Toutenburg, 1990). A total of 1000 new data sets with the observed and imputed scores were generated. The range of imputed values was limited to the range of observed values of the variables. Time series and autocorrelation plots of the worst linear function were performed to monitor the convergence of the generated imputation algorithms (Schafer, 1997). Sensitivity analyses were performed using only cases with available data; no significant differences were detected in any of the reported results.

A descriptive analysis was done of sample characteristics (i.e. age and SMMSE summary scores) for the two caregiver groups. Using the Student *t*-test, the live-in and non-live-in caregivers were compared in relation to their demographic characteristics, the Zarit total score with the three subscales from this outcome including negative appraisal, level of adequacy and burden strain, and the CES-D-10 and STAI-6 scales. Using Pearson’s correlation coefficient (*r*), the level of correlation between Zarit summary score (and three subscales), CES-D-10, and STAI-6 scales were analysed. Using LME regression analyses the two groups of caregivers (live-in or non-live-in) and demographics factors were compared in relation to each of the three scales (Zarit, CES-D-10, and STAI-6; https://cran.r-project.org/web/packages/lme4/lme4.pdf).

We performed a PCA using Oblimin rotation to establish the structure of the Zarit Burden Interview in this analytic sample of caregivers. We applied the Kaiser–Meyer–Olkin test to confirm suitability of the data for PCA. We visually inspected a screen plot to establish the number of factors.

## Results

### Participants and descriptive data

There were a total of 495 participants, including 240 in the live-in group and 255 in the live-elsewhere group. The mean age of the whole analytic sample was 61.64 (SD = 13.32) years. Participants in the live-in group were on average 68.60 (SD = 13.25) years old whereas participants in the live-elsewhere group were on average 55.10 (SD = 9.55) years old. The age difference between the groups was statistically significant (*p* < .001). The mean degree of disease severity for the whole analytic sample of care recipients was 17.88 (SD = 6.84) SMMSE points. Care recipients of caregivers in the live-in group had an average score of 17.45 (SD = 7.19) SMMSE points, whereas the average score of the live-elsewhere group was 18.29 (SD = 6.49) SMMSE points. The between-group difference in the degree of disease severity was not statistically significant (*p*= 0.174).

### Outcome data

The outcome data for the whole analytic sample and for each group are presented in Table 1.

**Table 1.** Caregiver outcomes (*N* = 495) of live-in (*n* = 240) and live-elsewhere (*n* = 255) in means and SD.

Outcome	Total sample	Live-in Caregivers	Live-elsewhere	p*	MD (95% CI)
Zarit	29.23 ± 16.35	31.68 ± 17.17	26.92 ± 15.21	**.0012**	**4.76 (1.91, 7.61)**
Negative appraisal caring^a^	14.20 ± 8.17	15.88 ± 8.19	12.61 ± 7.84	**<.0001**	**3.27 (1.86, 4.68)**
Adequacy ^a^	3.63 ± 3.03	3.37 ± 2.94	3.87 ± 3.10	.068	−0.5 (−1.03, 0.03)
Burden strain^a^	7.21 ± 5.97	7.68 ± 6.34	6.77 ± 5.59	.091	0.91 (−0.14, 1.96)
CES-D-10	9.80 ± 6.74	10.94 ± 6.42	8.73 ± 6.87	**.00025**	**2.21 (1.04, 3.38)**
STAI-6	40.55 ± 14.99	41.45 ± 14.69	39.71 ± 15.25	.199	1.74 (−0.90, 4.38)

* Students t-test.

^a^ Subscales for the ‘Zarit summary score’: higher scores on the Adequacy Scale and Negative Appraisal Scale indicate lower levels of adequacy and greater negative appraisal, respectively.

MD: mean difference; SD: standard deviation; CES-D-10: Center for Epidemiologic Studies Depression Scale; STAI-6: State-Trait Anxiety Inventory.

Alpha level = 0.05. Values in bold indicate that the *p* value is below the alpa level.

**Figure 1.**
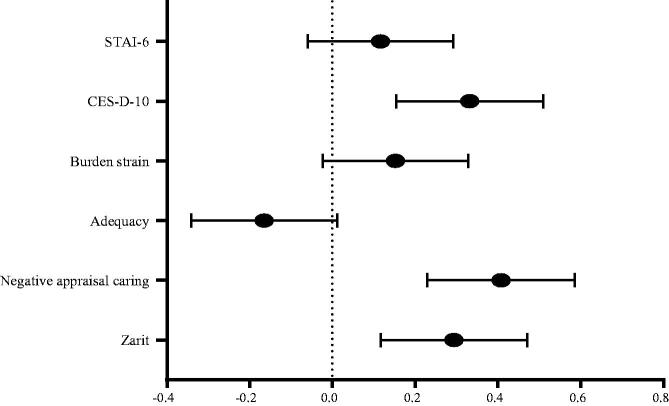
Mean difference in Edges’s *g* between between live-in and live-elsewhere caregivers on each outcome. Error bars represent 95% confidence intervals. The horizontal stagged line represents the line of no difference at an alpha level of .05. Live-in caregivers are on the right side of the horizontal line whereas live-eslewhere caregivers are on the left side.

In Figure 1, we show a non-weighted forest plot visualizing the standardized mean differences in Hedges’ *g* between caregivers living with the care recipient and the caregivers living elsewhere for each outcome.

### Main results

The comparison between the two groups assessed with Student *t*-tests indicated that live-in caregivers had significantly higher scores in the Zarit summary scores, in the Negative Appraisal scale, and in the CES-D-10 scale (Figure 1; Tables 1 and 2). There were no other significant between-group differences in any of the other outcomes (Figure 1; Tables 1 and 2). In addition, Pearson’s correlations between the total burden score and anxiety and depression were stronger in caregivers living-in. For example, Zarit *vs.* CES-D-10 (living-in: *r* = 0.665 *vs.* living-elsewhere: *r* = 0.566), Zarit *vs.* STAI-6 (living-in: *r* = 0.556 *vs.* living-elsewhere: *r* = 0.527). There was a stronger correlation between the CES-D-10 *vs.* STAI-6 for carers living-elsewhere (living-in: *r* = 0.705 vs. living-elsewhere: *r* = 0.726). Demographic factors were assessed in the LME models across each of the three outcome summary scores. Neither caregivers’ age nor SMMSE summary score moderated the relationship between caregiver living status and any of the outcomes.

**Table 2.** Linear mixed-effects regression models examining the relationship between caregivers’ status and each outcome.[Q]

	Zarit summary			CES-D-10 summary			STAI-6 summary		
	Estimate (β)	SE	*p*	Estimate (β)	SE	*p*	Estimate (β)	SE	*p*
Carer status (Live-elsewhere)	−17.393	8.089	**.032**	−2.628	0.694	**.0002**	−1.731	1.348	.199
**Covariate:**									
Carer age	−0.412	0.189	**.030**	−0.061	0.095	.522	−0.110	0.212	.605
SMMSE summary scores	−0.223	0.507	.660	−0.170	0.210	.419	−0.339	0.472	.473
**Interactions:**									
Carer age*Carer status	0.176	0.132	1.332	−0.005	0.055	.928	−0.092	0.123	.456
Carer age*SMMSE score	0.001	0.008	.874	0.002	0.003	.528	0.007	0.007	.341
**Model fit indices:**	*R*^2^ = 0.033, *F* = 4.344, RSE = 16.08			*R*^2^ = 0.022, *F* = 3.242, RSE = 0.022			*R*^2^ = 0.005, *F* = 1.474, RSE = 14.96		

SE: standard error; SMMSE: Standardized Mini–Mental State Examination; CES-D-10: Center for Epidemiologic Studies Depression Scale; STAI-6: State-Trait Anxiety Inventory; RSE: relative standard error.

Alpha level = 0.05. Values in bold indicate that the *p* value is below the alpa level.

## Discussion

Evidence indicates that caregivers living with the care recipient with dementia experience greater psychological burden and poorer mental health than those not living with the care recipient (Conde-Sala et al., 2010; Kim et al., 2012; Raccichini et al., 2009; Reed et al., 2014; Viñas‐Diez et al., 2017). However, some of these studies employed relatively small sample sizes and did not specifically assess differences in depression and anxiety between caregivers cohabiting with the care recipient and those living elsewhere (Conde-Sala et al., 2010; Kim et al., 2012; Raccichini et al., 2009; Reed et al., 2014; Viñas‐Diez et al., 2017). In this study, we aimed to address the limitations of previous studies and to compare caregivers living with the dementia care recipient to those living elsewhere on burden, depression, and anxiety by analyzing a large sample of caregivers from the ATTILA trial (Leroi et al., 2013).

Our results show that caregivers living with the care recipient with dementia experience greater psychological burden than those living elsewhere, thus supporting our first hypothesis. These findings are in line with previous smaller studies (Conde-Sala et al., 2010; Kim et al., 2012; Raccichini et al., 2009; Reed et al., 2014; Viñas‐Diez et al., 2017). Caregivers living with the care recipient also perceived their own caregiving role more negatively than caregivers living elsewhere but did not report greater burden strain or adequacy in providing care. Results also support in part the second hypothesis that caregivers living with the care recipient exhibited higher depression scores than caregivers living elsewhere (Conde-Sala et al., 2010; Kim et al., 2012; Raccichini et al., 2009; Reed et al., 2014; Viñas‐Diez et al., 2017). Neither caregivers’ age nor the care recipients’ level dementia severity moderated any of the observed relationships. This is in contrast to previous studies which showed higher levels of disease severity in dementia care recipients were associated with greater caregivers’ burden and poorer mental health (Cuijpers, 2005; Richardson et al., 2013; Watson et al., 2019). This may be due to the challenging nature of caring for someone with severe cognitive impairment (Watson et al., 2019) and may also reflect differences in the severity of dementia between this study and previous research.

Our results did not indicate that caregivers living with the care recipient showed greater anxiety than caregivers living elsewhere. Although the forest plot (Figure 1) did show the overall tendency for the live-in group to have greater burden and poorer mental health, levels of burden strain and anxiety were not different bettween the two groups. Although there was no between-group difference in adequacy of caregiving, it is interesting to observe that this was the only outcome which tended to favor the living-elsewhere group (Figure 1). This may be because caregivers living elsewhere felt that their ability to deliver care was insufficient to the needs of the care recipient as they were not available at all times. While some of these outcomes were not significant, it is possible the lack of between-group significance was due to low statistical power (Cumming, 2014).

### Limitations

Since we could not control for several key demographic factors, it is possible that participants living with the care recipient differed in important demographics factors relative to caregivers living elsewhere, which could explain the observed differences. For example, depression is more prevalent among women than in men (Eid, Gobinath, & Galea, 2019; Girgus, Yang, & Ferri, 2017), and female caregivers of dementia report higher levels of depressive symptoms than male caregivers (Watson et al., 2019). If the live-in group included more females than males relative to the other group, then the overall baseline risk for depression in the live-in group might have been higher. However, because we did not have data on caregivers’ sex, we could not examine whether sex moderated the relationship between caregiver status and each outcome.

We also could not examine whether being a spouse to the care recipient relative to being the next of kin (e.g. son/daughter) moderated the relationship between caregiver living status and burden. Occupying a lifelong relationship with the care recipient could affect burden differently than caring as a sibling or son/daughter. Also, since dementia is an umbrella term without a specific etiology (Vinters, 2015), we could not assess whether different types of dementia moderated the observed relationship between caregivers living status and burden as well as depression. This might be an important factor to consider because time spent and costs of caring likely differ depending on the type of dementia (Costa et al., 2012). Some caregivers such as spouses living and caring for a person with dementia may experience social isolation which could negatively affect their mental health (Egilstrod, Ravn, & Petersen, 2019). We did not have data on social isolation and therefore could not examine the potential moderating effects of social isolation on caregivers’ burden and mental health. Finally, since this study is cross-sectional, we cannot establish cause and effect and as such, our findings should be treated as hypothesis-generating only.

### Interpretation

Institutionalization is costly to the public purse as well as to private individuals (Luppa et al., 2010) and people with dementia often prefer to live at home in order to maintain social networks and quality of life (Mahler et al., 2014). Greater burden and poorer mental health in caregivers are associated with early institutionalization in the care recipient with dementia (Dorenlot, Harboun, Bige, Henrard, & Ankri, 2005; Stall et al., 2019). However, having a co-resident caring for someone with dementia is associated with a 20 times lower likelihood of institutionalization across one year (Banerjee et al., 2003). Without the support that informal caregivers provide to dementia patients, the care recipients would be most likely institutionalized earlier, and national economies would not be able to cope with the costs of community or residential care (Brodaty & Donkin, 2009; WHO, 2012). To delay institutionalization in people with dementia, it is important to generate strategies that can alleviate caregiver burden and improve their mental health.

Our data suggest that the caregivers who appear to be under the most psychological pressure are those who cohabit with the person with dementia. Focusing support on this group of caregivers may be appropriate given their relative perceived burden and their role in reducing institutionalization (Banerjee et al., 2003). Alleviating the need to cohabit with the care recipient by sharing caregiving responsibility among other family members, for example, may offer one useful strategy for mitigating the negative impact associated with living with the care recipient (Rivera, Bermejo, Franco, Morales‐González, & Benito‐León, 2009). Interventions to alleviate burden and improve mental health in caregivers for people with dementia are widespread (Vandepitte, Van Den Noortgate, Putman, Verhaeghe, Faes, et al., 2016; Vandepitte, Van Den Noortgate, Putman, Verhaeghe, Verdonck, et al., 2016). Although some of these strategies (e.g. psycho-educational interventions) may not always delay institutionalization in the care recipient (Vandepitte, Van den Noortgate, Putman, Verhaeghe, Faes, et al., 2016; Vandepitte, Van Den Noortgate, Putman, Verhaeghe, Verdonck, et al., 2016), tailoring strategies to mitigate caregivers’ burden by also targeting contextual factors may generate greater improvements in caregivers’ psychological and mental health outcomes.

Caregivers who seek respite often do so by contacting health services late in the care recipient’s natural history of the disease, at which point, providing respite to the caregiver may not be any longer useful (Vandepitte, Van Den Noortgate, Putman, Verhaeghe, Faes, et al., 2016; Vandepitte, Van Den Noortgate, Putman, Verhaeghe, Verdonck, et al., 2016). Targeting caregivers who cohabit with the care recipient at the time of dementia diagnosis,for example by generating a plan that involves sharing caregiving duties among family members (Rivera et al., 2009) and provides respite to the primary cohabiting caregiver (Vandepitte, Van Den Noortgate, Putman, Verhaeghe, Faes, et al., 2016; Vandepitte, Van Den Noortgate, Putman, Verhaeghe, Verdonck, et al., 2016), may enable the caregiver living with the care recipient to cope with the ongoing burden (Viñas‐Diez et al., 2017) more efficiently.

### Generalizability

Baseline data from the ATTILA trial were collected for a pragmatic randomized controlled trial the results of which can be applied across different settings thereby increasing generalizability. The pragmatic selection criteria used for sampling the care recipients reflect the extent to which the results for the primary outcome in the ATTILA trial as well as for the secondary outcomes in the current study can be generalized more broadly to clinical practice. For example, the caregivers were participants caring for individuals with dementia whose degree of disease severity extended across several SMMSE points including those occupying mild, moderate, and severe stages of dementia as well as memory complaints indicative of dementia. Disease severity is a relevant factor because routine clinical practice will receive patients with varying levels of cognitive impairment ranging from subjective cognitive decline to mild cognitive impairment to more severe forms of dementia with different underlying etiologies. Also, the caregivers’ cohort included participants from a wide spectrum of ages from young adults in the early 20 s and seniors in the early 80 s. Caregivers in the community tend to occupy different age groups including adult-children and older spouses or partners of the care recipient.

### Conclusion

In this study, we showed that caregivers living with the care recipient experienced greater psychological burden and poorer mental health than caregivers living elsewhere. Caregivers living with the care recipient may not be able to experience the same level of respite from caregiving duties compared to caregivers living elsewhere. It is possible that the unremitting provision of care may not allow a live-in caregiver to occasionally detach from caregiving duties while living elsewhere may provide an outlet for coping with the ongoing burden. Our findings are relevant to researchers and clinicians because they stress the significance of considering caregivers’ living arrangements when developing psychosocial interventions to mitigate caregivers’ burden.
